# 2-Aminoacetophenone as a potential breath biomarker for *Pseudomonas aeruginosa *in the cystic fibrosis lung

**DOI:** 10.1186/1471-2466-10-56

**Published:** 2010-11-07

**Authors:** Amy J Scott-Thomas, Mona Syhre, Philip K Pattemore, Michael Epton, Richard Laing, John Pearson, Stephen T Chambers

**Affiliations:** 1 University of Otago, Christchurch School of Medicine and Health Sciences, Department of Pathology, P.O. Box 4345, Christchurch, 8140, New Zealand; 2 Leipzig University, Department for Inner Medicine, Neurology and Dermatology, Johannisallee 32, 04103 Leipzig, Germany; 3 Canterbury District Health Board, Private Bag 4710, Christchurch, 8140 New Zealand

## Abstract

**Background:**

*Pseudomonas aeruginosa *infections are associated with progressive life threatening decline of lung function in cystic fibrosis sufferers. Growth of *Ps. aeruginosa *releases a "grape-like" odour that has been identified as the microbial volatile organic compound 2-aminoacetophenone (2-AA).

**Methods:**

We investigated 2-AA for its specificity to *Ps. aeruginosa *and its suitability as a potential breath biomarker of colonisation or infection by Solid Phase Micro Extraction and Gas Chromatography-Mass Spectrometry (GC/MS).

**Results:**

Cultures of 20 clinical strains of *Ps. aeruginosa *but not other respiratory pathogens had high concentrations of 2-AA in the head space of *in vitro *cultures when analysed by GC/MS. 2-AA was stable for 6 hours in deactivated glass sampling bulbs but was not stable in Tedlar^® ^bags. Optimisation of GC/MS allowed detection levels of 2-AA to low pico mol/mol range in breath. The 2-AA was detected in a significantly higher proportion of subjects colonised with *Ps. aeruginosa *15/16 (93.7%) than both the healthy controls 5/17 (29%) (p < 0.0002) and CF patients not colonised with *Ps. aeruginosa *4/13(30.7%) (p < 0.001). The sensitivity and specificity of the 2-AA breath test compared to isolation of *Ps. aeruginosa *in sputum and/or BALF was 93.8% (95% CI, 67-99) and 69.2% (95% CI, 38-89) respectively. The peak integration values for 2-AA analysis in the breath samples were significantly higher in *Ps. aeruginosa *colonised subjects (median 242, range 0-1243) than the healthy controls (median 0, range 0-161; p < 0.001) and CF subjects not colonised with *Ps. aeruginosa *(median 0, range 0-287; p < 0.003)

**Conclusions:**

Our results report 2-AA as a promising breath biomarker for the detection of *Ps. aeruginosa *infections in the cystic fibrosis lung.

## Background

**Figure 1:**
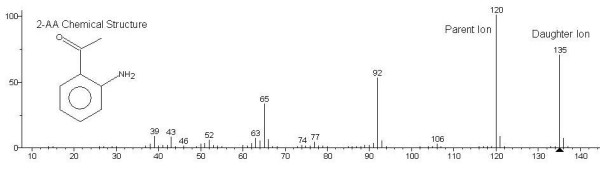
**NIST library formula and mass spectra of 2-AA under EI conditions**.

*Pseudomonas aeruginosa *is a Gram negative bacterium that produces a sweet "grape-like" odour during growth. In 1966 Mann [1] identified this compound as 2-aminoacetophenone (2-AA) by thin-layer chromatography. Cox & Parker [2] confirmed 2-AA as the compound responsible for this odour and we have also successfully detected and identified 2-AA in the headspace of *in vitro *cultures using gas chromatography/mass spectrometry (GC/MS) (Figure 1). 2-Aminoacetophenone is a small, volatile aromatic compound with a molecular weight of 135 g/mol (Figure 1). It is an intermediate product in the biosynthesis of quinazolines, which branches from the tryptophan catabolic pathway [3]. 2-Aminoacetophenone is consistently produced by multiple *Ps. aeruginosa *strains, on all culture media, and is a major metabolite [2-4], but the biological significance of this compound is unknown [2]. There is a single report of 2-AA detected in the headspace of *Escherichia coli *cultures [5] but 2-AA is not known to be produced by other respiratory pathogens. Because of the well described evidence of the production of 2-AA by *Ps. aeruginosa *and its apparent specificity we thought it may be useful as a volatile biomarker for infection and/or colonisation of the lung.

Cystic Fibrosis (CF) is an autosomal recessive disease affecting 1 in 3300 live Caucasian births [6]. The secretion of hyperviscous mucus in the CF-affected lung provides *Ps. aeruginosa *with a nutritionally rich growth environment, where it often grows to high cell densities (>10^9 ^cells/ml sputum) [7,8]. Although many other bacterial species persist and grow in the CF lung, chronic *Ps. aeruginosa *infection correlates with declining lung function and high mortality rates [4,9].

None of the current methods for detecting *Ps. aeruginosa *infections in CF patients are satisfactory. The culture of broncho-alveolar lavage fluid (BALF) is currently the reference standard for lower respiratory tract colonisation and infection [10]. However, this procedure is invasive and requires general anaesthetic in the very young. While BALF is not routinely performed, it has been used successfully in screening studies of both newly diagnosed and very young CF patients [11,12]. Cultures of sputum samples are significantly less sensitive than BALF [13] and cultures of the oropharynx have poor sensitivity and do not reliably predict the presence of organisms in the lower airways [14].

We have therefore undertaken an investigation to determine whether 2-AA is consistently produced by *Ps. aeruginosa *and other respiratory pathogens during *in vitro *culture and if 2-AA is detectable in the breath of CF patients colonised with *Ps. aeruginosa*.

## Methods

### Materials

2-Aminoacetophenone, hexamethyldisilazane and 1 L glass sampling bulbs were purchased from Sigma Aldrich, St. Louis, Missouri, United States of America. 2 L Tedlar^® ^bags were purchased from SKC Inc., PA, USA. Vials of tobramycin (Hospira, Wellington, New Zealand) and colistin (Link, Australia) for clinical use were obtained from the Christchurch Hospital Pharmacy, New Zealand.

### Solid Phase Micro Extraction

SPME fibres (60 μm Carbowax™/PEG) (Supelco, Bellefonte, Pennsylvania, United States of America) were used to pre-concentrate 2-AA from the headspace of cultures and breath samples. Each SPME fibre was preconditioned in a hot injector at 250°C, a test chromatogram was recorded and the clean and activated fibre was then exposed into the sample headspace.

### Detection of 2-AA by GC/MS

**Figure 2:**
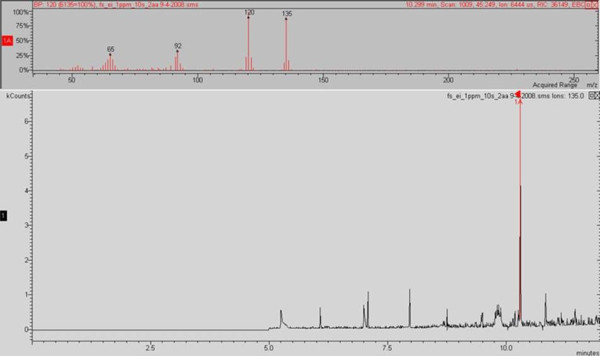
**Experimental spectra for 2-AA, obtained under GC/MS EI conditions, from *in vitro Ps. aeruginosa *cultures**.

A Saturn 2200 system (Varian, Palo Alto, USA) was used to perform the GC/MS analysis. A Zebron ZB-Wax 30 m × 0.25 mm × 0.25 mm (Phenomenex, Auckland, New Zealand) was connected into a Programmable Temperature Vaporiser (PTV-1079) injector. The temperatures of the injector, ion trap, manifold and transfer line were 250, 200, 60 and 250°C, respectively. The oven program commenced at 60°C for 2 minutes and was raised to 250°C at a rate of 10°C/min, at which the temperature was maintained for a further 2 minutes. Helium flow was set at a constant rate of 1.2 ml/min. The split vent was opened to a ratio of 1:50 after 1 min. Initial full scan spectra were obtained with a mass to charge (m/z) range of 40-650 utilising electron impact (EI) (Figure 2). A change to chemical ionisation (CI) with methanol as the precursor ion in full scan mode increased sensitivity for 2-AA in comparison to EI.

**Figure 3:**
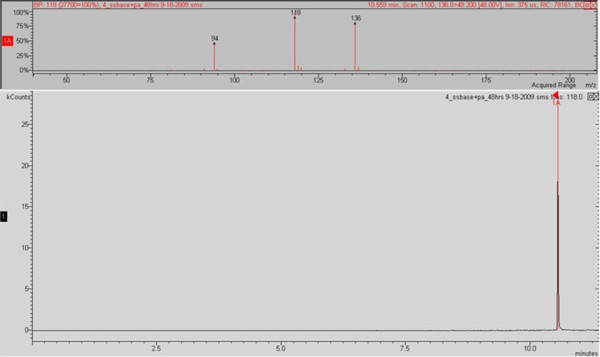
**Experimental spectra for 2-AA, obtained under GC/MS-MS CI conditions, from *in vitro Ps. aeruginosa *cultures**.

**Figure 4:**
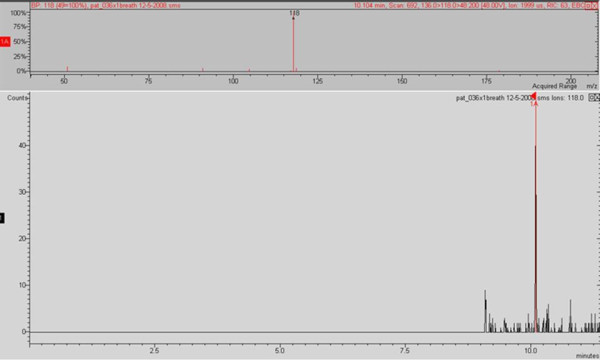
**Experimental spectra for 2-AA, obtained under GC/MS3 CI conditions, from a breath samples positive for 2-AA**.

In order to analyse 2-AA in breath it was necessary to utilise the MS-MS capabilities of the ion trap due to matrix and very low detection level requirements. Ion preparation for MS-MS analysis was in CI mode; the selected parent ion was m/z 135 with an isolation window of m/z 3; excitation storage level was 65; excitation amplitude was 48; the resulting MS-MS spectra featured three main peaks at m/z 118 (100%), m/z 136 (90%) and m/z 94 (45%) (Figure 3). Further sensitivity was achieved by additional fragmentation using MS^3^. The selected daughter ion was m/z 118 with an isolation window of m/z 3; excitation storage level was 65; excitation amplitude was 48; the resulting MS^3 ^spectra featured one main peak at m/z 118 (100%) (Figure 4).

### Micro-organisms

The following species were tested; *Ps. aeruginosa *(20 strains), *E. coli *(3 strains)*, Burkholderia multivorans *(3 strains), *Haemophilus influenzae *(3 strains), *Legionella pneumophila *(3 strains), *Moraxella catarrhalis *(3 strains), *Pseudomonas fluorescens *(3 strains), *Staphylococcus aureus *(3 strains), *Streptococcus pneumoniae *(3 strains) and *Aspergillus fumigatus *(3 strains). All strains were isolated and identified from a variety of respiratory specimens by Canterbury Health Laboratories, Christchurch, New Zealand by standard techniques [15]. *B. multivorans *strains were identified by API 20 NE and 16S rRNA sequencing [16]. All strains were stored at 4°C and re-plated regularly.

### Culture methods

All micro-organisms were cultured in 50 ml screw top bottles (Alltech Associates Inc. Illinois, United States of America) containing 10 ml of medium. Columbia Sheep Blood agar, Sheep Blood agar, and Mueller-Hinton agar were purchased from Fort Richards Laboratories Ltd, Auckland, New Zealand. Luria Bertani agar and M9 Minimal Media broth was prepared in the laboratory as specified in the Handbook of Microbiological Media [17]. The open screw cap contained a Teflon lined silicone septa. Two replicates of each micro-organism strain were inoculated into the media separately (0.5 MacFarlane Standard). The bottles were sealed tightly and incubated for at least 24 hours at 37°C, aerobically. The headspace gas was sampled using SPME by penetrating the septum.

### Drug testing for 2-AA

The agents were reconstituted into a glass vial with a Teflon septum according to the manufacturer's recommendations. The SPME fibre was introduced into the headspace through the septum for 5 minutes and then desorbed into the GC/MS instrument for analysis by full scan and MS-MS.

### Environmental air sampling

Levels of environmental 2-AA were determined from two sites where breath samples were collected (Respiratory Ward and Out Patients at Christchurch Hospital, New Zealand). Ten clean glass bulbs were prepared for environmental air sampling with a vacuum of 550 mm Hg. Bulbs were transported to sampling sites and the stop cocks opened releasing the vacuum and in turn sampling the environmental air. As soon as the sample was obtained the stopcocks were closed and the bulbs transported back to the laboratory for analysis. SPME fibres were inserted via the septa within 30 minutes. All sampling devices were incubated at 37°C for 24 hours before analysis.

### Subjects

Written informed consent was obtained from all subjects. Control subjects were healthy, non-smoking adults who had no known respiratory disease, and were not taking prescription or over the counter medication. Subjects (both adults and children) with clinically stable CF were identified by staff of specialist respiratory services at Christchurch Hospital, New Zealand. All subjects had CF diagnosed either by genetic analysis or at least three positive sweat tests (>60 mmol/L) and progressive obstructive airway disease characterised by a persistent, productive cough, hyperinflation of the lung fields on chest radiograph, and obstructive pulmonary function tests. Computerised medical records were reviewed for the past medical history and laboratory investigations and the diagnoses were confirmed by a specialist respiratory physician. CF patients with *Ps. aeruginosa *had to have at least two positive sputum and/or BALF tests for *Ps. aeruginosa *for inclusion into this group. The micro-organisms present in the respiratory secretions of CF patients were identified by culture at Canterbury Health Laboratories, Christchurch, New Zealand. A drug and dietary history was taken using a standardised questionnaire and entered into a data sheet.

### Breath sampling

Sampling was done between 8.30-9.30 am or 1.30-3.30 pm and the subjects food and/or fluid ingestion, over the 12 hours prior to breath collection, was recorded. Breath samples were obtained more than two hours after consumption of any foods.

All healthy controls and subjects with CF were studied in facilities sourced from a common air conditioning system. No other filters or control systems were used. Breath samples were collected by forced expiration of five breaths into the 1 L silanized glass sampling bulb without a nose clip or saliva trap. Stop cocks at each end were opened while breath was collected by forced expiration directly into the bulb without any additional mouthpiece. Some rest was allowed between breaths but all breath samples were collected within two minutes. As soon as the breath samples were collected the stop cocks were closed and the device was ready for analysis. Collection bulbs were taken to the GC/MS laboratory within 30 minutes of collection, SPME fibres were inserted via the septa within two hours. All sampling devices were incubated at 37°C for 24 hours before analysis.

### Statistical analysis

Degradation of 2-AA in the glass bulb was tested with the I-test adapted for unequal variances. A five parameter log logistic (5PL) model was fitted to the data and the EC_50 _estimated using the R package drc [18]. The 5PL model may be parameterised by equation 1 where *c *and *d *are the minimum and maximum concentrations respectively, *d *the hill slope, *e *the EC_50 _and *f *the asymmetry parameter.

(1) $$Concentration=c+\frac{d-c}{{\left[1+{\left(\frac{time}{e}\right)}^{b}\right]}^{f}}$$

The four parameter log logistic (4PL) model has the same parameterisation as the 5PL with *f *set to 1, forcing symmetry about the point of inflection. ANOVA shows that the 5PL had significantly better fit to the data than the 4PL model (p < 0.0002). The estimated minimum concentration for the 5PL model was -3.18 (SE 8.99) hence we fitted a 5PL model with the minimum concentration, *c*, constrained to 0 to aid interpretability. Table 1 shows the Akaike Information Criterion (AIC) for each model and the estimated EC_p _where the 95% confidence intervals were estimated by the delta method [18]. The AIC is a measure of fit which penalises additional parameters; lower values indicate better fit so the table shows that the 5PL model fits better than the 4PL while fixing the minimum concentration provides a marginal improvement. The colonised subjects were compared with both control groups for presence versus absence (below threshold of 2-AA detection) in breath with Fisher's exact test. The Mann-Whitney test was used to compare the peak integration values of the three group's breath tested. Statistical tests and modelling was performed in R version 2.10 [19].

**Table 1:** 2-AA degradation models

Model	AIC	**EC**_**50**_	95% CI	
5PL c = 0	144.84	11.73	10.94	12.52
5PL	146.48	12.38	9.03	15.73
4PL	162.30	9.71	8.92	10.49

### Ethical approval

Ethical approval for the study was obtained from the Upper South Island Ethics Committee, New Zealand and the study was conducted in accordance with the standards for clinical research of the University of Otago, New Zealand.

## Results

### 2-AA production by bacteria *in vitro*

Twenty clinical isolates of *Ps. aeruginosa *(including 12 mucoid strains) obtained from a variety of respiratory samples submitted to Canterbury Health Laboratories were tested. 2-Aminoacetophenone was detected in the head space in "high" quantities (in the milli mol/mol range) in all isolates after 24 hours incubation on Sheep Blood agar. 2-Aminoacetophenone was detected 10 out of 10 times from the head space of a single *Ps. aeruginosa *strain cultured on Columbia Sheep Blood agar. This same strain also produced 2-AA when cultured on Mueller-Hinton agar, Luria Bertani agar, M9 Minimal Media broth and laboratory tap water. 2-Aminoacetophenone was not detected from the head space of *H. influenzae*, *L. pneumophila*, *M. multivorans*, *Ps. fluorescens*, *B. multivorans*, *S. pneumoniae *and *A. fumigatus *strains upon culturing on Columbia Sheep Blood agar, Sheep Blood agar and Mueller-Hinton agar. *S. aureus *and *E. coli *strains did not produce 2-AA when grown on Sheep Blood agar or Mueller-Hinton agar. However, one out of three *S. aureus *strains and two out of three *E. coli *strains produced trace levels of 2-AA (low pico mol/mol) on Columbia Sheep Blood agar.

### 2-AA stability in breath collection systems

**Figure 5:**
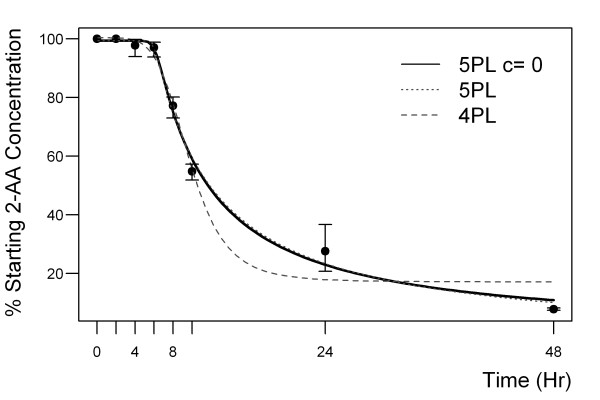
**A time degradation graph of 2-AA in glass sampling bulbs**. The 2-AA levels (mean and SD) from three replicate experiments are shown and a five parameter logistic function fitted to the degradation curve (11.73 hours (95% CI 10.94, 12.52). 2-AA levels at 8 and 10 hours were significantly less (P < 0.001) than four and six hours.

2 L Tedlar^® ^bags and 1 L deactivated glass bulbs were spiked (10 μmol) with 2-AA reference standard and were analysed for stability of 2-AA at 37°C. Within two hours of spiking, only 10% of the initial concentration was detectable in the Tedlar^® ^bags and no 2-AA was detectable after 24 hours. 2-Aminoacetophenone was found to be stable over two hours in the glass sampling bulb and after 24 hours approximately 40% was still present. The preliminary comparison showed the superiority of the glass sampling bulbs for 2-AA. To determine the "half-life" of 2-AA in the glass sampling bulb system, 2-AA was spiked into the glass bulbs (three replicates) at a concentration of 10 μmol. The resulting time degradation is illustrated in Figure 5. The EC_50 _for 2-AA was estimated to be 11.73 hours (95% CI 10.94, 12.52). A peak integration area of 88 was determined as the lower limit of detection (detection limit) for 2-AA in the 1 L glass sampling bulb filled with ambient air. This peak integration value was determined to be a concentration 50 pmol/mol (n = 15 with a standard deviation of 4.8 pmol/mol).

### Environmental air sampling

Air samples were analysed from the two collection sites in MS^3 ^mode to ensure no environmental contamination of breath samples with 2-AA. The levels of 2-AA detected in the environmental samples were well below the threshold of detection in all samples analysed from both sites.

### Study population

The clinical characteristics, medication use and micro-organisms isolated from CF sputum samples are shown in Tables 2, 3 and 4. As expected, subjects colonised with *Ps. aeruginosa *were associated with older age, more complications of CF with more anti-pseudomonal and antimicrobial therapy. *S. aureus *colonisation was more frequent in those who were not colonised with *Ps. aeruginosa *11/13 (85%) compared to those that were colonised with *Ps. aeruginosa *7/16 (43%). Food and fluid intake in the previous 12 hours was similar in both the subject and the control group (Table 5).

**Table 2:** Characteristics of subjects included in breath sampling

	Healthy controls	CF - *Ps. aeruginosa *colonised	CF - *Ps. aeruginosa *non-colonised
Number (n)	17	16	13
Age years (mean range)	38 (25-54)	26.9 (13-59)	14.9 (6-43)
Male (%)	38%	63%	53%
BMI (kg/m^2^) (mean range)		19.07 (17.1-24.4)	19.9 (14.8-37.2)
Respiratory function: FEV_1_%		44.92 (13-93)	77.15 (48-99)
FVC%		59.51 (1.14-102)	91.1 (55-120)
Genetic analysis		Sweat test (3)	Sweat test (3)
		ΔF508/ΔF508 (9)	ΔF508/ΔF508 (5)
		ΔF508/G542X (3)	ΔF508/G542X (1)
		ΔF508/c.1339delA (1)	c.F508/c.1766+1g (1)
			ΔF508/3849+10kbct (1)
			c.1624g > t(p.g542x)/c.1
			624g > t(p.g542x) (1)
			ΔF508/P67L (1)
Diagnoses^^^			
Pancreatic insufficiency		15	12
Chronic rhinosinuitis		9	0
Osteopenia		8	0
Gastro-oesophageal disease		7	0
CF related diabetes		4	3
Vitamin A, D & E deficiency		5	1
Asthma		5	0
Allergic bronchopulmonary		0	3
aspergillosis			
Distal intestinal obstruction		1	2
syndrome			
Previous tuberculosis		1	0
Pancreatitis		1	0
Chronic nasal polyps		0	1
Haemoptysis		0	0
Chronic eczema & ichthyosis		1	1

^^^Some CF subjects had multiple diagnoses

**Table 3:** Medication prescribed to cystic fibrosis patients at time of breath sampling*

Medication	CF - *Ps. aeruginosa *colonised (n = 16)	CF - *Ps. aeruginosa *non-colonised (n = 13)
Creon^®^	8	13
Tobramycin nebulised	9	1
Colistin nebulised	7	1
Fluticasone propionate	3	5
Salbutamol	4	4
Budesonide-formoterol	4	3
Itraconazole	2	5
Dornase alfa	3	1
Budesonide	3	0
Ciprofloxacin	0	3
Salmeterol	2	0
Beclomethasone	2	0
Vancomycin	1	0
Formoterol fumarate	1	0
Amphotericin B	0	1

* All CF subjects were taking multiple medications

**Table 4:** Micro-organism isolated from sputum of patients with cystic fibrosis^#^

Organism	CF - *Ps. aeruginosa *colonised	CF - *Ps. aeruginosa *non-colonised
	(n = 16)	(n = 13)
*Pseudomonas aeruginosa*	16	0
*Staphylococcus aureus*	7	11
*Aspergillus fumigatus*	6	2
*Haemophilus influenzae*	0	8
Oropharyngeal flora	5	3
*Candida *species	1	2
*Burkholderia multivorans*	0	2
*Inquilinus limosus*	1	0
*Penicillium *species	0	1
*Stenotrophomonas maltophilia*	0	1
*Scedosporium apiospermum*	1	0
*Wangiella dermatitidis*	1	0

^# ^All CF subjects had multiple micro-organisms in the lung

**Table 5:** Survey of food consumption prior to 12 hours of breath collection

Food	Healthy controls (n = 17)	CF - *Ps. aeruginosa *colonised (n = 16)	CF - *Ps. aeruginosa *non-colonised (n = 13)
Milk	10	7	9
Cheese	1	2	0
Energy Drink	1	2	2
Coffee	6	3	1
Yoghurt	2	4	1
Green tea	0	1	0
Beer	0	0	0
Sweet corn	0	0	0
Corn chips	0	1	1
Honey	0	2	0

### Detection of 2-AA in breath samples

**Figure 6:**
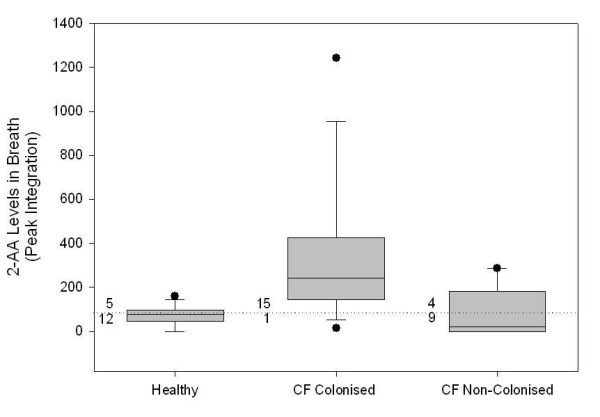
**2-AA detection in breath**. The peak integration values for 2-AA were significantly higher in CF patients colonised with *Ps. aeruginosa *than in CF patients not colonised (p < 0.001) with Ps. aeruginosa and healthy subjects (p < 0.0002). The numbers of subjects with detectable 2-AA in each group are shown. The lower limit of detection (peak integration of 88) is shown as a dotted line.

The 2-AA was detected in a significantly higher proportion of subjects colonised with *Ps. aeruginosa *15/16 (93.7%) than both the healthy controls 5/17 (29%) (p < 0.0002) and CF patients not colonised with *Ps. aeruginosa *4/13 (30.7%) (p < 0.001). The sensitivity and specificity of the 2-AA breath test compared to isolation of *Ps. aeruginosa *in sputum and/or BALF was 93.8% (95% CI, 67-99) and 69.2% (95% CI, 38-89) respectively. The peak integration values for 2-AA analysis in the breath samples are shown in Figure 6. These values were significantly higher in the breath of patients colonised with *Ps. aeruginosa *(median 242, range 0-1243) than the healthy controls (median 0, range 0-161; p < 0.001) and CF subjects not colonised with *Ps. aeruginosa *(median 0, range 0-287; p < 0.003).

## Discussion

There is well-established and growing interest in the detection and identification of micro-organisms by measuring their release of volatile organic compounds (VOCs) [20]. Because 2-AA is associated with the "grape-like" smell of *Ps. aeruginosa *and is more consistently produced than the common pyocyanin [21], it was hypothesized that 2-AA could be a potential breath biomarker for *Ps. aeruginosa *growth in the CF lung. We have confirmed that *Ps. aeruginosa *consistently produces 2-AA during *in vitro *growth on several types of media. Our analytical GC/MS technique also detected trace amounts of 2-AA in two of the three *E. coli *strains tested and in one of the three *S. aureus *strains, however, the level was two magnitudes lower than the 2-AA levels detected in *Ps. aeruginosa *cultures. Although *Ps. aeruginosa *produces millimol levels of 2-AA in ideal aerobic conditions, we have yet to determine whether 2-AA production is dependent on the growth phase of the organisms or if it is affected by oxygen tension or nutrient supply. It is possible these conditions will differ between early lung colonisation and established *Ps. aeruginosa *infection, potentially affecting the performance of a breath test.

Our breath sampling system involves the collection of breath into a transportable device. Here, preliminary data showed that significant levels of background emissions from the Tedlar^® ^bag itself (even after N_2 _flushing) do interfere with the 2-AA detection. Even in the deactivated glass sampling bulb 2-AA does not remain stable due to its reactive, polar chemical nature (11.73 hours (95% CI 10.94, 12.52)). 2-Aminoacetophenone is thought to undergo oxidation to 2-nitroacetophenone but can be analysed accurately provided breath samples are processed promptly. A five parameter logistic function was fitted to the degradation data (Figure 5) and used to estimate EC_50_. Such models do not assume symmetry and it has been shown that any function with less than five parameters is unlikely to have the flexibility necessary to produce a high-quality fit to asymmetric sigmoidal dose-response data [22].

The analysis of specific markers for infectious diseases in breath is difficult due to the very low levels of volatiles present in the breath matrix [23,24]. However, GC/MS^3 ^is a very sensitive technique and coupled with SPME pre-concentration extremely low levels of analytes can be detected. Comparing spiked samples (ambient air spiked with the 2-AA reference standard) with real breath samples can be used for an assessment of a detection range provided the same experimental procedure is followed.

The most important finding of this study was that 2-AA was present above the limit of detection in a higher proportion in the breath of subjects with CF who were colonised with *Ps. aeruginosa*, than both control groups (Figure 6). The peak integration values of 2-AA can be regarded as semi-quantitative only, but are consistent with the results of the analysis for presence or absence of 2-AA in breath, and support the conclusion that the likely source of the 2-AA are *Ps. aeruginosa *colonising the lungs. In contrast there was no evidence that *S. aureus *colonisation of the lung produced a positive 2-AA breath test despite trace amounts being detectable in the headspace of *in vitro *cultures. This preliminary study supports the concept that 2-AA may potentially serve as a biomarker of pseudomonas infection or colonisation in CF patients. This is consistent with previous studies of *Aspergillus *colonisation and *Mycobacterium tuberculosis *infection showing that specific biomarkers of these organisms are detectable in the breath by GC/MS techniques [24-26]. 2-Aminoacetophenone was also detectable in a small proportion of both healthy controls and subjects with CF who did not have evidence of *Ps. aeruginosa *on sputum sampling. The levels of 2-AA were very low and the origins of this signal are, at present, unknown.

As with any breath test, food, environment and medication are potential sources of the diagnostic marker. 2-Aminoacetophenone has been reported in foods and beverages namely corn, dairy, honey products and wine [27-29]. Breath testing immediately after the consumption of any one of these products may potentially give a false positive result. Investigation into whether ingested 2-AA rich food and beverages may increase 2-AA levels in the breath is currently ongoing. While ingested 2-AA may reach the breath it is possible that some or all may be metabolised by the host. It is not known whether prescribed or over the counter medication may also artificially increase or decrease the levels of 2-AA found in the breath but we have tested clinical preparations of tobramycin and colistin to exclude these as a potentially confounding source of 2-AA in this study. It is possible that some medications may breakdown to 2-AA which will require detailed investigations given the wide variety of medication taken by CF sufferers. A further possible source of 2-AA, particularly in CF patients is microbial colonisation of the mouth which was not detected on the sputum sampling. This may come from oral flora as well as *Ps. aeruginosa*. Further studies are needed to exclude these possible sources and it may be possible to reduce 2-AA levels in healthy individuals by careful control of the experimental conditions.

One of the major problems with breath testing in general is the sampling technique. Only a small number of studies have been performed to investigate the factors that affect the concentration of endogenous VOC's on the breath before it exits the mouth [30]. While deactivated glass bulbs have been used successfully in previous studies [24-26] the most appropriate breath sampling technique for microbial derived VOCs has not yet been determined.

## Conclusions

In conclusion our investigation supports the concept that 2-AA is a potential breath biomarker for *Ps. aeruginosa *and warrants further investigation as an indicator of *Ps. aeruginosa *colonisation or infection in the CF lung.

## Competing interests

The authors declare that they have no competing interests.

## Authors' contributions

AST completed the experiments testing the stability of 2-AA in the current breath collection system. AST collected and analysed environmental air samples and breath samples, participated in the design of the study and drafted the manuscript. MS set up the GC/MS protocols for the detection of 2-AA and also completed the detection of 2-AA in bacterial cultures *in vitro*. PP participated in recruiting paediatric CF patients for breath analysis and in the final layout of the manuscript. ME was involved in study design and analysis of the result. ME also helped further improve the final manuscript draft. RL participated in recruiting adult CF patients from his clinics for breath analysis and the final layout of the manuscript. JP was integral in not only the study of the designs but also for the statistical analysis of all the results presented in this manuscript. SC participated in the design of the study, collection and analysis of clinical data, co-ordination of Respiratory Practitioners and the writing of the manuscript. All authors read and approved the final manuscript.

## Pre-publication history

The pre-publication history for this paper can be accessed here:

http://www.biomedcentral.com/1471-2466/10/56/prepub
